# Tissue Regeneration without Stem Cell Transplantation: Self-Healing Potential from Ancestral Chemistry and Physical Energies

**DOI:** 10.1155/2018/7412035

**Published:** 2018-07-03

**Authors:** Federica Facchin, Eva Bianconi, Silvia Canaider, Valentina Basoli, Pier Mario Biava, Carlo Ventura

**Affiliations:** ^1^Department of Experimental, Diagnostic and Specialty Medicine (DIMES), University of Bologna, Via Massarenti 9, 40138 Bologna, Italy; ^2^National Laboratory of Molecular Biology and Stem Cell Bioengineering of the National Institute of Biostructures and Biosystems (NIBB) - Eldor Lab, Innovation Accelerator, CNR, Via Piero Gobetti 101, 40129 Bologna, Italy; ^3^Department of Biomedical Sciences, University of Sassari, Viale San Pietro 43/B, 07100 Sassari, Italy; ^4^Scientific Institute of Research and Care Multimedica, Via Milanese 300, 20099 Sesto San Giovanni, Italy

## Abstract

The human body constantly regenerates after damage due to the self-renewing and differentiating properties of its resident stem cells. To recover the damaged tissues and regenerate functional organs, scientific research in the field of regenerative medicine is firmly trying to understand the molecular mechanisms through which the regenerative potential of stem cells may be unfolded into a clinical application. The finding that some organisms are capable of regenerative processes and the study of conserved evolutionary patterns in tissue regeneration may lead to the identification of natural molecules of ancestral species capable to extend their regenerative potential to human tissues. Such a possibility has also been strongly suggested as a result of the use of physical energies, such as electromagnetic fields and mechanical vibrations in human adult stem cells. Results from scientific studies on stem cell modulation confirm the possibility to afford a chemical manipulation of stem cell fate *in vitro* and pave the way to the use of natural molecules, as well as electromagnetic fields and mechanical vibrations to target human stem cells in their niche inside the body, enhancing human natural ability for self-healing.

## 1. Introduction

The human body continuously regenerates due to the peculiar properties of its resident stem cells.

These cells possess the unique ability to self-renew and differentiate, and the balance between these two processes defines the stem cell fate and their primary role in tissue regeneration [1].

Regeneration is the recovery of the organ structure and function after injury and it is at the basis of our self-healing potential and therefore of the preservation of human health. Such a process exhibits remarkable grading in the way it is fashioned in living organisms, since, within the same species, the regenerative potential is different among the various organs [2].

To rescue damaged tissues and restore functional organ mass, huge efforts have been made in the growing field of regenerative medicine, engaging scientific research in the understanding of the molecular mechanisms through which the regenerative potential of stem cells (as human mesenchymal stem cells - hMSCs) may be unfolded into a clinical application [3]. Stem cells in fact have the capability to differentiate into a wide range of adult cells and the discovery and isolation of them paved the way to new hopes in the regenerative field.

On the other hand, many aspects of the cell-based therapy prevent the use of stem cells to regenerate organs and tissues: among them, a large amount of stem cells is required and the senescence process occurs during primary cell expansion. Moreover, it is not easy to isolate stem cells and to commit all of them toward a specific phenotype, since they can differentiate in all types of mature cells, including cancer cells. Therefore, a proper set up of *in vitro* MSC expansion, cryopreservation, and banking should be necessary to establish safety and efficacy in transplanted patients.

In addition, most of the applications of stem cells directed on patients are still under the phase of experimental trials, except for some procedures actually used in clinical practice, as the bone marrow transplantation in hematology [4].

Even tissue engineering, one of the branches of the regenerative medicine based upon tissue regeneration from cells with the aid of biomaterials and growth factors, still is facing several problems. In fact, the regenerated tissues usable by patients are still very limited, as skin, bone, cartilage, capillary, and periodontal tissues [5]. Moreover, the engineered artificial tissue still exhibits some limitation correlated to the dimensions of the construct that cannot be used for the recovery of serious defects. Actually, the only amenable engineered tissues with a tridimensional structure are vases, cave structures like the trachea [6], or tissues which are not physiologically scattered, since the viability of cells seeded on a scaffold gradually decreases with thickness. Even the use of growth factors alone or in association with 3D constructs is still considered as being not completely safe since the resulting influence on recipient's environment remains in part to be established. Finally, other hurdles remain, such as finding the best scaffold, the most suitable bioreactor, and the optimal solution for seeding different cell populations in order to have a relevant mature material implantable on patients.

All these issues need to be addressed before cells or engineered constructs can be used routinely in the clinical setting. Therefore, multiple studies have long been running to address the modulation of relevant physiological traits known to be involved in tissue homeostasis and in the activation of the stem cell niches. To this end, besides investigating the effects of synthetic molecules, many researchers have also focused their attention on the effects elicited by natural molecules and physical energies. Their findings are reported below.

## 2. Natural Molecules

The ability to regrow body parts is common to a lot of animal species, although the regenerative potential varies among taxa [7]. Some phyla are able to rebuild every part of the body, while others cannot regenerate internal organs [8].

*Danio rerio* (zebrafish) is among the organisms capable of amazing regenerative processes, prompting the needs for uncovering the underlying regeneration strategies. Zebrafish is since recently commonly used as an animal model of organogenesis and regeneration, owing to its ability to regenerate complex organs, like the heart, the central nervous system, and the limbs, at an extraordinarily higher efficacy than humans [2, 3, 9–16]. Another species exhibiting astonishing regenerative potential is the Mexican axolotl (*Ambystoma mexicanum*), which can make self-copies regenerating a missing limb, tail, or parts of the brain, heart, and lower jaw. Other creatures in the spotlight for their regenerative capabilities are the salamanders, as well as several frogs [17], or the tunicates [18]. Despite their evolutionary distance, as in the case of zebrafish which is separated by about 450 million years from humans, our stem cells can still sense ancestral microenvironmental cues from these species, as shown by the finding that human cord blood CD34^+^ cells are recruited into early vasculogenesis upon transplantation in pre-gastrulation, but not post-gastrulation zebrafish embryos [19]. Akin to this view is the finding that conserved transcriptional responses have been discovered among the differentiation of hMSCs, Xenopus embryogenesis, and axolotl regeneration, identifying common networks across model species that are associated with depolarization (changes in cellular resting potential) [20].

On the whole, these findings and the deployment of comparative biology into the analysis of conserved evolutionary patterns in tissue regeneration may lead to the identification of natural molecules capable to extend their regenerative potential from ancestral species to human tissues through the manipulation of common/similar mechanisms in their resident stem cells.

Investigation of the role of natural molecules in stem cell biology is becoming a growing area of inquiry. Psoralidin, for example, a natural phenolic compound found in the seeds of *Psoralea corylifolia*, has been seen to inhibit NOTCH1 in breast cancer stem cells and in breast cancer cells, leading to a growth arrest and inhibition of epithelial to mesenchymal transition (EMT) [21]. Moreover, two herbal extracts (*Tithonia diversifolia* leaf extract and *Momordica foetida* extract) led to a decrease of the adipogenesis and accumulation of lipid droplets in human adipose-derived stem cells (hADSCs) [22, 23]. Two natural compounds, honokiol (a low-molecular-weight polyphenol isolated from the genus *Magnolia*) and hyperoside (a flavonoid compound extracted from *Hypericum perforatum*), were shown to potentially induce the differentiation into neurons in the murine embryonic carcinoma cell line P19 [24]. Synthetic compounds created by the assembly of natural molecules have also been proven effective in the modulation of stem cell biology *in vitro* and *in vivo*. To this end, mixed esters of naturally occurring molecules, such as hyaluronan mixed esters with butyric and retinoic acids (HBR), have been shown to remarkably increase cardiogenesis and vasculogenesis in mouse embryonic stem cells [25] and hMSCs [26], enhancing the ability of term placenta hMSCs of promoting the regeneration of infarcted myocardium *in vivo* in both small (rat) and large (pig) animal models with post infarct heart failure [27, 28]. Intriguingly, in the myocardium of infarcted rats, HBR itself acted through the intracellular release of its natural grafted molecules to afford significant decrease in infarct size, and apoptotic myocytes, leading to reverse myocardial remodeling, normalization in myocardial contractility, and increase in vital myocardial mass and metabolism, through the enhancement/recruitment of the number of endogenous stro-1 (a mesenchymal stem cell marker)-positive stem cells, the increase in the number of local elements with pericyte identity and important revascularization processes [29]. This finding shows the feasibility of chemical targeting damaged organs to afford tissue survival and repair without stem cell transplantation. Consonant with these results, a simple cocktail of hyaluronic, butyric, and retinoic acids was able to improve islet graft revascularization and function by adipose tissue-derived hMSCs in diabetic rats [30].

The addition of melatonin to this mixture of natural molecules was able to shift the commitment of hMSCs towards an osteogenic fate, indicating the feasibility of creating a multicomponent, multitarget ensemble of natural agents to chemically redirect the multilineage repertoire of hMSCs [31].

A major breakthrough in the effort of using natural arrays of molecules to drive cellular fates under normal and pathological conditions came by the discovery that extracts from zebrafish embryos obtained at different developmental stages were able to counteract the proliferation rate of several cancer cell lines [32–35]. Extracts from the beginning, intermediate and final embryonic development stages led to an evident increase in p53 expression in association with the growth reduction [33]. In some cancer cell lines, such as kidney adenocarcinoma, the proliferation decrease was associated with changes in pRb phosphorylation, a cell cycle modulator [34]. Moreover, in colon adenocarcinoma cells, an activation of the p73-dependent apoptotic pathway was observed [35]. A mixture of early, middle, and late developmental stage zebrafish extracts was also able to enhance cell survival to toxic stimuli, as shown by the reduction in mortality observed in cells from mouse hippocampal slices (CA1 area) that had been subjected to serum deprivation or NMDA (N-methyl-D-aspartate) treatment [36]. These findings and previous observations showing that embryonic microenvironment is able to suppress tumor development during cell differentiating processes [37, 38] led us to further investigate whether zebrafish embryonic factors may also be exploited in a developmental stage manner to control essential features in stem cell dynamics. To this end, we successfully used early-stage developmental zebrafish extracts (obtained from 5.15 hours post fertilization embryos) on early-passage hADSCs to enhance the stem cell expression of multipotency, and the transcription of *TERT*, encoding the catalytic subunit of telomerase, as well as the gene expression of *BMI1*, a chromatin remodeler acting as a major telomerase-independent repressor of senescence [39].

On the whole, the above mentioned studies, showing the possibility to afford a chemical manipulation of stem cell fate *in vitro*, may pave the way to the use of natural or synthetic chemistry to target human stem cells where they are already resident in all body tissues. This would lead to the development of a regenerative medicine executed without the needs for (stem) cell or tissue transplantation.

## 3. Physical Energies

The possibility of using physical energies to boost regenerative processes has been strongly suggested by the ability of electromagnetic fields and mechanical vibrations to drive efficient *in situ* reprogramming of the differentiating and regenerative potential of our endogenous stem cells.

We are in fact embedded in a wide variety of physical stimuli, including electromagnetic fields, light radiation, and mechanical oscillatory patterns. In this sense, our life which contains a seeming infinity of rhythmic oscillations, including calcium and pH intracellular oscillations [40–42], as well as the rhythmic expression of genes and proteins [43, 44], can be considered as a part of the vibratory nature of the universe.

It is now evident that our cells perceive and generate energies like magnetic fields and mechanical oscillations [45–47]. Cells contain a network of microtubules that, due to their electrical polarity and intrinsic vibration modes, is able to generate high-frequency electric fields with radiation features [48]. Applying scanning tunneling microscopy (STM) to microtubules growing onto a nanoelectrode array, within an artificial cell replica designed to pump electromagnetic frequencies, has shown the existence of resonance patterns between the tubulin dimers, or the whole microtubules, and the applied frequencies [49]. STM also provided evidence that such resonance patterns could be imaged as specific “tunneling current profiles” corresponding to the pumped electromagnetic frequencies [49]. The frequency region selectivity for engaging particular types of conformational modifications establishes that pure mechanical changes can be remotely managed in an atomically way by using electromagnetic fields.

The importance of the microtubule network as an *information-transporting-system* is also deduced by the finding of multilevel memory-switching properties in a single brain microtubule [50]. Even DNA, despite its role of storage and expression of genetic information, when considered as an electrically charged vibrational entity, may contribute to cell polarity, also by virtue of its constant assembly into different loops and domains that are an essential component of the nanomechanics and nanotopography imparted to this macromolecule by transcription factors and molecular motors. Accordingly, electromagnetic resonance frequency spectra have been revealed for DNA, which was found to exhibit electromagnetic resonances in the wide frequency range from KHz, MHz, GHz, to THz [51].

Recently, regenerative medicine has been focused on the use of biophysical stimuli to modulate cellular dynamics [52]. Physical factors in the cellular microenvironment, including matrix mechanics, cell geometry and shape, mechanical forces, and nanotopographical aspects of the extracellular matrix, can modulate the stem cell fate [53, 54]. There is evidence that this type of regulation is highly affected by coexisting insoluble, adhesive, mechanical, and topological cues contained and dynamically regulated within the stem cell niche [55, 56]. Biophysical stimuli can be sensed and transduced into intracellular biochemical and functional responses by stem cells, a process known as mechano-transduction [55]. The stem cell sensory machinery can at the same time perceive and integrate several signals from the niche and turn them into coherent responses affording downstream modulation of gene expression and stem cell fate [55, 57–59].

For years, scientists tried to drive stem cell fate by the aid of chemistry, increasing cell proliferation with growth factors or fabricating 3D constructs derived from the combination of stem cells or mature adult cells, with natural or artificial polymers. Only in the last years, efforts have been made to interact with cells *in vivo*, directly on patients or on animal models, and *in vitro* on cell cultures. Recently, some research groups have shown the possibility to use physical stimuli directly on patients, tissues, and cells [60].

The idea behind the use of physical stimuli on tissues and body was already proposed in 1974 by Richard Nuccitelli who gained evidence on endogenous ionic current and interaction with electric field in multicellular animal tissues [61]. Nowadays, it is possible to explain changes in cellular behavior, following electromagnetic stimulation, considering an effect on cell polarity [62] and on the stem cell niche in the body [63].

The use of physical energies for therapeutic purpose is now well known, being approved by the Food and Drug Administration (FDA) and used on patients. Several devices based on different physical mechanisms have been designed, and the beneficial effects have been observed directly on patients. Ultrasounds have been used for medical purposes since the 1950 in some pathological situations, such as tendinitis or bursitis [64].

Even the use of extremely low-frequency electromagnetic fields (ELF-EMFs) with frequencies lower than 100 Hz, and magnetic field intensity spanning from 0.1 to 20 mT, became a useful therapy for soft tissue regeneration, fracture repair, and osteoporosis treatment [65]. The mechanisms of action of ELF-EMFs are not clear yet. However, it has been shown that electric currents can accelerate cell activation [66] and influence epigenetic remodeling. In particular, the use of 50 Hz ELF-EMF on GC-2 cells decreased genome-wide methylation and the expression of DNA methyltransferases [67] in neural stem cells (NSCs) isolated from the hippocampus of newborn mice. Moreover, the ELF-EMF irradiation at 1 mT, and 50 Hz, for 12 days enhanced NSC proliferation and neuronal cell fate specification through Cav1 channel-dependent regulation and histone modification [68]. These results show the feasibility of using physical stimuli to affect cell fate.

Within this context, we have first demonstrated the possibility to use ELF-EMFs to modulate the gene transcription of essential growth regulatory peptides in adult myocardial cells [60] and to enhance cardiogenesis and terminal differentiation into spontaneously beating myocardial cells in mouse embryonic stem (ES) cells [69]. Then, by the aid of a radio electric asymmetric conveyer (REAC), we found that properly conveyed radioelectric fields of 2.4 GHz could produce important biological effects in mouse ES cells and human adult stem cells. In both cell types, we showed that REAC-conveyed radioelectric fields elicited an increase of the expression of stemness-related genes, followed by the commitment towards neuronal, myocardial, and skeletal muscle lineages [70, 71]. The same differentiating outcomes were induced by REAC exposure in human skin fibroblasts [72]: for the first time, human non-stem somatic adult cells were committed to lineages in which they would never otherwise appear. This effect was mediated by a biphasic change in pluripotency gene expression, a temporary overexpression followed by a down regulation, and did not require the use of viral vector-mediated gene transfer technologies or cumbersome synthetic chemistry.

Noteworthy, REAC exposure of hADSCs was able to turn stem cell senescence, occurring after prolonged (up to 30 passage) *in vitro* expansion, into a reversible phenomenon, associated with a decrease in the expression of senescence-associated *β*-galactosidase and an increase in *TERT* gene expression and telomere length. The REAC action also enhanced the gene transcription of *BMI1* and that of stemness-related genes, establishing a telomerase-independent arm for senescence reversal [73]. These findings may have important biomedical implications, since senescent stem cells decrease their self-renewal and differentiation potential, reducing their ability for tissue regeneration *in vivo* and the possibility of a prolonged expansion *in vitro* prior to transplantation.

Compounding the wide-ranging biological effects of REAC stimulation is the observation that this technology was able to promote neurological and morphofunctional differentiation in PC12 cells [74], a rat adrenal pheochromocytoma cell line displaying metabolic features of Parkinson's disease. Cell response to the electromagnetic field was mediated by the transcriptional activation of neurogenic genes, as *neurogenin-1*, *β3-tubulin*, and *nerve growth factor (NGF)*, and was associated with a consistent increase in the number of cells expressing both *β*3-tubulin and tyrosine hydroxylase [74]. These findings open the new perspective of using physical energies in the treatment of neurodegenerative diseases and in the reprogramming of cancer (stem) cells into normal regenerative elements. More recently, we found that the REAC action could be significantly counteracted by stem cell treatment with 4-methylumbelliferone (4-MU), a potent repressor of type-2 hyaluronan (HA) synthase and endogenous HA synthesis [75]. This observation suggests that REAC-mediated responses may have occurred through an essential pleiotropic role of this glycosaminoglycan in regulating (stem) cell polarity.

Extracorporeal shock waves (ESW) represent another type of biophysical stimuli that is increasingly being applied in the field of regenerative medicine and that could be classified as “mechanotherapy” (*i.e.*, extracorporeal shock wave therapy, ESWT). In fact, ESW are “mechanical” waves, characterized by an initial positive very rapid phase, of high amplitude, followed by negative pressure, producing a “microexplosion” that can be directed on a target zone (body, tissue, or cells) in order to stimulate or modify the cells in their behavior. Shock waves are generated by an electrohydraulic device that produces underwater high-voltage condenser spark discharge, conveyed by an elliptical reflector on tissues or cells.

In the 1980s, shock waves were used in urology (lithotripsy) to disintegrate renal stones [76]. Then, ESW application has been extended to other fields, showing promising hopes for promoting tissue healing and the recovery from pathological disorders. One of the first applications was in the orthopedic field, in order to induce neovascularization and improve blood supply and tissue regeneration. Investigations on the use of this technology spread progressively, and leading to its application in the treatment of musculoskeletal disorders [77], tendon pathologies [78], bone healing disturbances, and vascular bone diseases [79]. The use of ESW has also been extended to the field of dermatology for the wound healing disturbances and ulcers. However, to date, the exact mechanism through which cells convert mechanical signals into biochemical responses is not well understood yet. Emphasis has been placed so far into mechanisms mediated by ATP release and P2 receptor activation that may foster cell proliferation and tissue remodeling via Erk1/2 activation [80, 81], as well as PI-3K/AKT and NF-*κ*B signaling pathways, and the implication of TLR3 signaling and subsequent TLR4. Several studies performed *in vitro* proved the effect of ESW on cell modulation through “mechano-transduction”. Recently, ESW were found to activate ADSCs through MAPK, PI-3K/AKT, and NF-*κ*B signaling pathways [82, 83] and to induce in HUVEC cells an overexpression of angiogenic factors and of caveolin-1, a constitutive protein of caveolae, implicated in the regulation of cell growth, lipid trafficking, endocytosis, and cell migration [84].

In addition, the ESWT effect on cell behavior proved to be a dose-dependent phenomenon. In a study published by Zhang and coworkers, cells exposed to low-energy ESW (0.04 e 0.13 mJ/mm^2^) improved the expression of some angiogenic factors, such as eNOS, Ang-1, and Ang-2. On the other hand, at higher energy, ESW induced a reduction in angiogenic factor expression and an increase in apoptosis [85]. These findings suggest that the biological effects of shock waves strongly correlated with the intensity of applied energy and thus with the related mechanical forces.

Recently, the effects of shock waves have been characterized on the expression of IL-6, IL-8, MCP-1, and TNF-*α* in human periodontal ligament fibroblasts [86]. Following an early inhibition on the expression of pro-inflammatory mediators, shock waves elicited a dose-related increase in IL-6 and IL-8, while down-regulating TNF-*α* expression [86]. Most of the literature showed an anti-inflammatory effect of ESWT *in vivo* [59, 78, 79, 87, 88]. Nevertheless, the pro-inflammatory effect of ESWT partially observed on cells *in vitro* may suggest a pro-activator event mediated by cytokine and chemokine expression. It was supposed that the shock wave impulses on cells were able to create a pro-inflammatory milieu, mediated by mechano-transduction [80]. However, this mechanism may involve a more complex action on the whole niche architecture, with the embedded (stem) cells behaving as sensors and activators of the regenerative response.

In actual fact, mechanical vibration may represent a relevant modality to affect stem cell reprogramming *in vivo* without having to resort to transplantation procedures. In this regard, we have shown and patented for the first time the cell ability to exhibit “vibrational” (nanomechanical) signatures of their health and their multilineage repertoire [89]. Wide-ranging vital processes are fashioned around the nanomechanical features of subcellular structures, like the microtubular networks, imparting feature characteristic of connectedness and synchronization that can be transferred and recorded from the cell surface. Atomic force microscopy (AFM) can be used to gain insights on cellular nanomechanical properties [89, 90], providing the chance to identify vibrational signatures that can be used to drive lineage-specific commitments in different stem cell populations *in vitro* or even *in vivo* to promote endogenous rescue in diseased organs.

## 4. Conclusion

The emerging view of a (stem) cell biology governed by physical forces and influenced by ancestral natural molecules may lead us to reinterpret the way we envision the field of regenerative medicine for a near future.

In fact, due to the diffusive nature of electromagnetic fields and mechanical vibrations, the chance is emerging to target and reprogram the stem cells where they are, enhancing our natural ability for self-healing without the needs for (stem) cell transplantation which still shows remarkable limitations.
